# Supplementary material for: Epidemiology, Risk Factors, and Outcomes of Opportunistic Infections after Kidney Allograft Transplantation in the Era of Modern Immunosuppression: A Monocentric Cohort Study
Source: J Clin Med. 2019 Apr 30;8(5):594. doi: 10.3390/jcm8050594 (PMC6572426; doi:10.3390/jcm8050594)
Supplement: Supplementary file 1 [file jcm-08-00594-s001.pdf]

Supplemental data

Journal of Clinical Medicine

Table S1. Allograft survival risk factors univariable analysis.

	With BK virus nephritis			Without BK virus nephritis		
Variables	HR	IC 95%	*p*-value	HR	IC 95%	*p*-value
**Recipient characteristics**						
Female	0.80	0.49–1.33	0.39	0.80	0.47–1.37	0.42
Age at transplantation	1.01	0.99–1.03	0.31	1.01	0.99–1.03	0.20
Dialysis	1.73	0.63–4.75	0.29	2.03	0.64–6.50	0.23
Hemodialysis	1.29	0.64–2.60	0.48	1.47	0.67–3.24	0.34
CMV+	1.15	0.60–2.20	0.66	1.23	0.60–2.50	0.57
HCV+	1.55	0.71–3.40	0.27	1.82	0.82–4.00	0.14
HIV+	0.67	0.16–2.72	0.57	0.76	0.18–3.11	0.70
Initial nephropathy						
Hypertension	1.15	0.55–2.41	0.71	1.33	0.63–2.79	0.46
Unknown origin	0.83	0.44–1.59	0.58	0.78	0.38–1.58	0.48
Diabetes	1.66	0.93–2.95	0.08	1.51	0.80–2.85	0.20
Genetic	0.50	0.21–1.15	0.10	0.57	0.25–1.33	0.19
Glomerulopathy	1.23	0.73–2.08	0.43	1.11	0.63–1.98	0.71
Tubular and interstitial	1.01	0.37–2.77	0.99	1.18	0.43–3.25	0.75
Urologic	0.68	0.21–2.16	0.51	0.80	0.25–2.56	0.71
Other	0.56	0.08–4.01	0.56	0.68	0.09–4.94	0.71
Diabetes before transplant	1.66	0.96–2.86	0.07	1.60	0.89–2.88	0.12
Diabetes after transplant	1.55	0.70–3.47	0.28	1.49	0.63–3.54	0.37
**Donor characteristics**						
Age	1.02	1.01–1.04	0.004	1.02	1.01–1.04	0.008
Expanded Criteria Donor	1.74	1.07–2.81	0.02	1.54	0.92–2.57	0.10
eGFR	1.00	0.99–1.00	0.63	1.00	0.99–1.01	0.95
Living donor	1.05	0.45–2.42	0.91	1.24	0.53–2.88	0.62
CMV+	1.72	1.04–2.85	0.03	1.86	1.07–3.20	0.03
**Sensitization risk factors**						
Anti HLA antibodies	1.01	0.63–1.63	0.96	0.98	0.59–1.63	0.93
Former kidney transplantation	0.62	0.20–1.98	0.42	0.72	0.22–2.29	0.57
Donor specific anti-HLA antibodies	1.63	0.87–3.06	0.13	1.79	0.92–3.46	0.09
**Combined Transplants**						
Pancreas	0.73	0.10–5.24	0.75	0.84	0.12–6.04	0.86
Liver	0.94	0.23–3.82	0.93	1.08	0.26–4.43	0.91
Heart	*	*	*	*	*	*
**Kidney Transplant Characteristics**						
Cold ischemia time	1.03	0.99–1.06	0.04	1.03	0.99–1.07	0.11
Induction Immunosuppressive regimen	1.00	0.31–3.20	0.99	0.89	0.28–2.85	0.84
Basiliximab	0.85	0.53–1.37	0.51	0.86	0.51–1.43	0.55
Antithymocyte glubulin	1.17	0.73–1.89	0.51	1.14	0.68–1.90	0.62
Intravenous Immunoglobulin	0.85	0.42–1.72	0.66	0.76	0.35–1.68	0.57
Rituximab	1.36	0.62–2.98	0.44	1.39	0.59–3.23	0.45
Maintenance immunosuppressive regimen						
Calcineurin inhibitors	*	*	*	*	*	*
Mycophenolate Mofetil	*	*	*	*	*	*
Steroids	*	*	*	*	*	*
Belatacept	*	*	*	*	*	*
**White blood cells at the time of transplant**						
Leucocytes (/1000)	0.97	0.87–1.09	0.64	0.95	0.84–1.07	0.66

Neutrophils (/1000)	1.00	0.89–1.13	0.96	0.97	0.85–1.11	0.70
Lymphocytes (/1000)	0.66	0.43–1.00	0.05	0.69	0.44–1.07	0.10
TCD4 cells (/1000)	0.17	0.06–0.51	0.001	0.18	0.06–0.59	0.004
TCD4 cells (%)	0.97	0.95–0.99	0.005	0.98	0.95–1.00	0.02
TCD8 cells (/1000)	0.47	0.15–1.54	0.21	0.52	0.14–1.85	0.31
TCD8 cells (%)	1.01	0.99–1.03	0.22	1.01	0.99–1.03	0.27
**Follow up**						
Opportunistic infection (OI) episode	3.04	1.65–5.61	0.0004	1.09	0.39–3.02	0.87
CMV viremia before OI	1.40	0.84–2.32	0.20	1.51	0.88–2.58	0.13
BK viruria before OI	1.41	0.83–2.39	0.21	*	*	*
BK viremia before OI	3.04	1.65–5.61	0.0004	*	*	*
BK virus nephritis	7.90	3.86–16.19	<0.0001	*	*	*
Acute rejection before OI	3.54	2.18–5.76	<0.0001	4.11	2.44–6.93	<0.0001
